# Utilization and affordability of health insurance coverage for rare disease drugs in a first-tier city in Northeast China from 2018 to 2021: a study based on the health insurance claims database

**DOI:** 10.1186/s12939-024-02225-0

**Published:** 2024-07-31

**Authors:** Yaqun Wang, Na Zhou, Baoxin Li, Zixuan Lv, Shengnan Duan, Xin Li, Ni Yuan

**Affiliations:** 1 School of Public Health, Dalian Medical University, Dalian, Liaoning China; 2 Department of Health Policy and Management, Peking University, Beijing, China; 3 Department of Regulatory Science and Pharmacoeconomics, School of Pharmacy, Nanjing Medical University, Nanjing, Jiangsu China

**Keywords:** Rare diseases, Orphan drugs, Utilization, Affordability, Accessibility

## Abstract

**Objective:**

The accessibility issue of orphan drugs in China is prominent. Based on real-world data from a tier-one city in Northeast China, this study aims to analyze the current usage and affordability of orphan drugs for rare diseases.

**Methods:**

The data was sourced from the health insurance claims data of a certain city from 2018 to 2021, including a total of 16 orphan drugs. The utilization of orphan drugs is assessed using four indicators: frequency of medical insurance claims, medication cost, defined daily doses (DDDs), and defined daily drug cost (DDDc). Affordability is measured using the concept of catastrophic health expenditure (CHE).

**Results:**

Between January 2018 and December 2021, there were a total of 2,851 medical insurance claims in the city, with a total medication costs of $3.08 million. Overall, during the study, there was a year-on-year increase in the utilization frequency of individual rare disease drugs in the city, with DDDs rising from 140.22 in 2018 to 3983.63 in 2021. Additionally, the annual medication costs of individual drugs showed a consistent upward trend, increasing from $10,953.53 in 2018 to $120,491.36 in 2021. However, the DDDc of individual drugs decreased from $398.12 in 2018 to $96.65 in 2021.The number of sales and the amount of sales for orphan drugs in community pharmacies have significantly increased. Prior to medical insurance coverage, out of the 16 orphan drugs, 9 drugs had annual treatment costs exceeding CHE for urban residents, and 15 drugs had annual treatment costs exceeding CHE for rural residents. After medical insurance coverage, there were no drugs with out-of-pocket costs exceeding CHE for urban residents, while 8 drugs had out-of-pocket costs exceeding CHE for rural residents. Furthermore, both before and after medical insurance coverage, the four treatment drugs for idiopathic pulmonary arterial hypertension were more affordable compared to the four treatment drugs for multiple sclerosis.

**Conclusion:**

The usage frequency of orphan drugs in a certain city increased gradually, but the disease burden remained heavy. More policy support should be provided to the priority rare disease populations, and the rare disease medical security and diagnosis and treatment systems should be improved.

## Background

Rare diseases, also known as “orphan diseases,” refer to diseases with a low incidence and relatively few affected individuals [1]. The World Health Organization (WHO) defines rare diseases as those with a prevalence of 0.65‰-1‰ among the total population [2]. Despite the relatively low incidence and prevalence of rare diseases, considering China’s large population, the absolute number of rare disease patients is not insignificant. This has significant implications for various aspects of society, economy, and healthcare, making rare diseases a public health issue that cannot be ignored. According to two batches of “Rare Disease Catalogs” jointly issued by the National Health Commission, Ministry of Science and Technology, Ministry of Industry and Information Technology, and other six ministries of the People’s Republic of China, a total of 207 rare diseases have been included so far [3, 4]. Medications for rare diseases, also known as “orphan drugs,” refer to drugs used for the prevention, treatment, and diagnosis of rare diseases [5]. The accessibility of orphan drugs in China is a prominent issue, with 95% of rare diseases lacking medication for treatment as of 2022, highlighting the critical importance of accessibility to orphan drugs [6]. Even in cases where treatment options are available, the high cost of orphan drugs leads to some patients foregoing treatment due to financial constraints [7].

Ensuring access to orphan drugs and reducing the financial burden on rare disease patients has been a key focus of China’s national healthcare system. The majority of general diagnostic and treatment services, as well as an increasing number of orphan drugs, have been included in the national reimbursement drug list (NRDL), effectively alleviating the medication burden on rare disease patients. Taking the world’s first drug successfully negotiated in China in 2021 for the treatment of the rare disease spinal muscular atrophy (SMA), nusinersen sodium injection, as an example, the price per bottle has been reduced from the original price of $108,500 to approximately $5,100 per bottle. This substantial price reduction has notably eased the economic burden on patients using this medication and allowed more rare disease patients to benefit from it.

To date, the NRDL has undergone seven significant adjustments. Starting from 2017, the national drug price negotiation (NDPN) policy has become an important method for NRDL adjustments, and a dynamic adjustment mechanism has been implemented since 2020. In terms of disease areas, access to products for anti-infection, tumors, and the blood system ranks high [8]. According to the report from the National Healthcare Security Administration (NHSA), from 2019 to 2023, the number of orphan drugs newly included in the NRDL through NDPN policy each year was 9, 6, 7, 7, and 13. However, in China, the inclusion of a drug in the NRDL does not necessarily mean that patients can immediately use it in hospitals. Therefore, the NHSA and the National Health Commission of the People’s Republic of China implemented the “dual-channel” management policy for negotiated drugs in 2021 [9, 10]. The “dual-channel” refers to the utilization of designated medical institutions and designated community pharmacies as two channels to meet the reasonable needs in terms of negotiated drug supply guarantee and clinical utilization, while being included in the medical insurance reimbursement mechanism. In China, the inclusion of a medication in medical insurance does not guarantee immediate access for patients in hospitals. During the hospital admission process, there are various policy restrictions and challenges, including total insurance expenditure limits, restrictions on the number of drugs in the hospital’s formulary, and drug cost ratios, all of which impact the implementation of negotiated drugs [11]. Since patients are unable to obtain medications directly from hospitals, the primary objective of the “dual-channel” policy is to address the accessibility issue for orphan drug patients. Thus, the implementation of the “dual-channel” policy can provide timely and effective benefits to patients. Since the implementation of these policies, the NHSA, as the policy maker and executor, along with the medical institutions as the primary responsible entities for the allocation and use of negotiated drugs, and the designated community pharmacies as the important entities for supplementary supply guarantee, have jointly ensured the implementation of the “dual-channel” approach for negotiated drugs at the national level [12].

Improving the accessibility of orphan drugs in China, bridging the “last mile” of patient medication, and reducing the disease burden on patients are crucial tasks for the improvement of China’s medical insurance system. Currently, research on orphan drugs mainly focuses on analyzing relevant policies and regulations, comparing domestic and international healthcare systems, and examining the market availability of orphan drugs [13–15]. There is limited research on studying the usage patterns and affordability of orphan drugs in a specific region in China using Real World Data (RWD). However, changes in indicators such as frequency of medical insurance claims, medication cost, defined daily doses (DDDs), defined daily drug cost (DDDc), and catastrophic health expenditure (CHE) can reflect the implementation effects of the NDPN and access policies, providing important reference for policy improvement and optimization. Therefore, the purpose of this study is to analyze the usage status of orphan drugs in a specific city in China, based on the implementation of the annual NDPN for orphan drugs. The study aims to provide empirical analysis and propose targeted recommendations for the issues encountered in the process of orphan drug utilization and affordability. This study utilizes health insurance claims data from a specific city to investigate the real-world application and accessibility of orphan drugs. It aims to provide empirical evidence for policymakers, promote the development and improvement of the orphan drug field, and enhance the support provided by the healthcare system for orphan drugs.

## Data and methods

### Data source and cleaning

The data for this study were sourced from the Healthcare Security Administration’s health insurance claims databases in a specific city, covering the period from January 2018 to December 2021. This city is located at the forefront of economic development and medical standards in the northeastern region of China, with a resident population of 6.087 million (registered population). The data consisted of two components: patient basic information and claims information. The patient basic information included variables such as individual code, date of birth, medical treatment category (outpatient/inpatient), and health insurance schemes. It is important to note that this study does not involve any personally identifiable information. The claims information included variables such as actual claims date, designated type (medical institution/community pharmacy), generic name of drug, drug unit price, medication cost, out-of-pocket medication cost, and reimbursed disease category.

The original database contained a total of 3,549 claim records. After excluding data with full self-payment (out-of-pocket medication cost-medication cost ≥ 0), 698 records were removed, resulting in a final dataset of 2,851 records, covering 16 different drugs. To minimize potential human errors during the data cleaning process, two independent researchers conducted the data cleaning and cross-checked the final results.

### Study population

This study focuses on 16 orphan drugs included in the NRDL between 2017 and 2020, examining their usage and affordability in the specific city from January 2018 to December 2021. There are a total of six medications in injectable form and a total of ten medications in oral sustained-release form (capsules or tablets). Among them, “Octreotide” was included in the special negotiation for anti-cancer drugs in 2018 and was successfully approved for market access. Its approved indications are “gastroenteropancreatic neuroendocrine " and “acromegaly.” Both of these indications were included in the second batch of the Rare Disease List of China in September 2023. Therefore, this medication was included in this study. “Infliximab” and “Vedolizumab” are registered for the rare disease indication of “Crohn’s disease.” Although this disease is not included in the Rare Disease List, epidemiological data from domestic and international sources show that “Crohn’s disease” meets the criteria for a rare disease. Moreover, in 2019 and 2020, the definition of rare diseases by the NHSA was not very clear. Therefore, these two medications were also included in the scope of this study. Table 1 provides basic information on the drugs.

**Table 1:** Basic information of the drugs

Serial number	Generic name of drugs	Dosage form	Registered indications for rare diseases	Marketing authorization holder	China market approval date	Inclusion in medical insurance negotiation date
1	Recombinant human interferon beta-1b	Injection	Multiple sclerosis	Bayer AG	2008	2017
2	Recombinant human coagulation factor VIIa	Injection	Hemophilia	Novo Nordisk A/S	2002	2017
3	Octreotide	Injection	Acromegaly、Gastroenteropancreatic neuroendocrine	Novartis Pharma Schweiz AG	2003	2018
4	Bosentan	Tablet	Idiopathic pulmonary arterial hypertension	Janssen-Cilag AG	2006	2019
5	Deferasirox	Tablet	Thalassemia	Novartis Pharma Schweiz AG	2010	2019
6	Riociguat	Tablet	Idiopathic pulmonary arterial hypertension	Bayer AG	2017	2019
7	Ruxolitinib	Tablet	Primary myelofibrosis	Novartis Pharma Schweiz AG	2017	2019
8	Macitentan	Tablet	Idiopathic pulmonary arterial hypertension	Actelion Pharmaceuticals Ltd	2017	2019
9	Selexipag	Tablet	Idiopathic pulmonary arterial hypertension	Actelion Pharmaceuticals Ltd	2018	2019
10	Teriflunomide	Tablet	Multiple sclerosis	Sanofi-aventis U.S. LLC	2018	2019
11	Infliximab	Injection	Crohn’s disease	Janssen Biologics B.V.	2007	2019
12	Siponimod	Tablet	Multiple sclerosis	Novartis Pharma Schweiz AG	2020	2020
13	Fingolimod	Capsule	Multiple sclerosis	Novartis Pharma Schweiz AG	2019	2020
14	Edaravone	Injection	Amyotrophic lateral sclerosis	Simcere Pharmaceutical Co.Ltd	2019	2020
15	Nintedanib	Capsule	Idiopathic pulmonary fibrosis、Systemic sclerosis	Boehringer Ingelheim International GmbH	2017	2020
16	Vedolizumab	Injection	Crohn’s disease	Takeda Pharma A/S	2020	2020

Note: Except for the failure to renew the recombinant human interferon beta-1b in the 2019 NDPN, the remaining 15 drugs are currently within the agreement period

### Research indicators

This study conducted a comprehensive evaluation of orphan drug utilization and affordability. To facilitate comparison among different drug varieties and specifications, this study followed the recommendations of the WHO and employed the defined daily dose (DDD) as the unit of measurement for drug dosage, using defined daily doses (DDDs) to reflect the clinical utilization of the drug. Therefore, the drug utilization was primarily assessed based on frequency of medical insurance claims, medication cost, DDDs, and defined daily drug cost (DDDc). Additionally, following the WHO/Health Action International (HAI) methodology for assessing medicine availability, the concept of catastrophic health expenditure was utilized to measure affordability [16]. The specific meanings of the indicators are as follows.

#### Drug utilization

“Frequency of medical insurance claims” refers to the number of claims submitted by patients to medical insurance providers within a certain period of time. The unit of measurement is “times.” It is commonly used to measure the frequency of drug usage by a patient within a specific period.

“Medication cost” refer to the costs incurred by patients when purchasing drugs before insurance reimbursement.

DDDs are indicators developed by the WHO to evaluate the actual consumption of drugs and trends in their usage. A higher DDDs indicates a higher frequency of drug usage and a greater clinical preference for that drug [17]. In addition, DDDs have additivity, allowing for the comparison of drug utilization frequencies between different classes of medications and at different stages of treatment. The DDD represents the average daily dose for adults for the main therapeutic purpose of the drug, as referenced in the “Guidelines for Diagnosis and Treatment of Rare Diseases (2019 Edition) [18]” and the drug package inserts approved by the National Medical Products Administration of China (NMPA).

DDDc represents an indicator of the daily cost for patients maintaining the dosage of an orphan drug. A higher value of DDDc indicates a heavier burden of daily medication costs for patients, which means they need to pay higher expenses to maintain the drug treatment.

$${\text{DDDs = }}\:\frac{{{\text{total}}\,{\text{sales}}\,{\text{quantity}}\,{\text{of}}\,{\text{drug}}\:}}{{DDD}}$$

$${\text{DDDc = }}\frac{{{\text{total}}\,{\text{medication}}\,{\text{costs}}\,{\text{of}}\,{\text{drug}}}}{{DDDs}}$$

#### Affordability of drugs

This study uses catastrophic health expenditure (CHE) as an indicator to assess the affordability of orphan drugs. CHE refers to the expenses borne by a household for healthcare services within a certain period, when the proportion of these expenses to the household’s non-food expenditure exceeds 40% [19]. When the cost of a medication exceeds the household’s CHE, the drug is considered unaffordable. In this study, the affordability of drugs is evaluated by calculating the multiple of the annual treatment cost of the drug to the resident’s CHE, where a multiple ≤ 1 indicates that the annual treatment cost of the drug is affordable. Per capita non-food expenditure of households = Household population ×(Per capita consumption expenditure - Per capita food, tobacco, and alcohol expenditure [20]). According to the Seventh National Population Census Bulletin of a certain city, the average household size in that city is 2.29 people. The annual per capita consumption expenditure and per capita food, tobacco, and alcohol expenditure of households in the city are obtained from the Statistical Bureau of the city. The per capita non-food expenditure of households in the city from 2018 to 2021 is shown in Table 2. The per capita non-food expenditure and average household size are calculated as average values based on the total residents of the city, including both urban and rural areas. The currency exchange rate between US dollar and Chinese Yuan was: USD1.0 = CNY6.4515 in 2021.

**Table 2:** Average per capita non-food expenditure of households in a certain city from 2018 to 2021

	2018	2019	2020	2021
**Urban**	$7659.27	$8083.63	$7462.70	$8876.09
**Rural**	$2894.42	$2958.77	$2823.64	$3385.49

### Research methods

This study employed Excel software to conduct a descriptive analysis of the usage, reimbursement, and affordability of rare disease drugs in a certain city from 2018 to 2021. The chi-square test was performed using SPSS software to analyze the frequency of medical insurance claims at different designated medical institutions among patients of different types. Kruskal-Wallis tests (referred to as “K-W tests” hereafter) were conducted to analyze the medication expenses among different types of patients. Statistical analysis was performed using SPSS 26 software.

## Results

### Analysis of frequency of medical insurance claims in different designated medical institutions among patients of different types

From Table 3, according to the results of the chi-square test, there were statistically significant differences in the choice of different levels of medical institutions among rare disease patients based on different medical treatment categories (*P* < 0.001), health insurance schemes (*P* < 0.001), claims years (*P* < 0.001), and dosage forms (*P* < 0.001).

**Table 3:** Comparative analysis of frequency of medical insurance claims for purchasing rare disease medications among different patient types at different designated types

Group	Designated type			Frequency of medical insurance claims (%)	*P*
	Secondary hospital	Tertiary hospital	Community pharmacy		
**Total frequency of medical insurance claims**	3(0.1%)	343(12.0%)	2505(87.9%)	2851(100.0%)	
**Medical treatment category**					*P* < 0.001
Outpatient	1(0.0%)	1(0.0%)	15(0.5%)	17(0.6%)	
Outpatient for major diseases and outpatient for chronic diseases	0(0.0%)	11(0.4%)	1(0.0%)	12(0.4%)	
Outpatient special drugs	0(0.0%)	190(6.7%)	2489(87.3%)	2679(94.0%)	
Inpatient	2(0.1%)	141(4.9%)	0(0.0%)	143(5.0%)	
**Health Insurance Schemes**					*P* < 0.001
URRBMI	2(0.1%)	73(2.6%)	449(15.8%)	524(18.4%)	
UEBMI	1(0.0%)	270(9.5%)	2056(72.1%)	2327(81.6%)	
**Claims year**					*P* < 0.001
2018	0(0.0%)	9(0.3%)	10(0.4%)	19 (0.7%)	
2019	0(0.0%)	80(2.8%)	105(3.7%)	185(6.5%)	
2020	0(0.0%)	137(4.8%)	631(22.1%)	768(26.9%)	
2021	3(0.1%)	117(4.1%)	1759(61.7%)	1879(65.9%)	
**Dosage form**					*P* < 0.001
Oral agents	0(0.0%)	4(0.2%)	1588(55.6%)	1592(55.8%)	
Injection agents	3(0.1%)	339(11.9%)	917(32.2%)	1259(44.2%)	

Note: Due to rounding, the sum of the percentages may not equal 1

In terms of medical treatment categories, patients primarily purchased orphan drugs through outpatient special drugs (94.0%), followed by inpatient settings (5.0%). Outpatient, outpatient for major diseases, and outpatient for chronic diseases combined accounted for only 1% of the total. Among patients seeking healthcare through outpatient special drugs, the majority purchased orphan drugs from community pharmacies (87.3%).

Regarding health insurance schemes, the frequency of Medical Insurance Claims for patients covered by urban-rural residents’basic medical insurance (URRBMI) was approximately one-fifth of that for patients covered by urban employees’basic medical insurance (UEBMI). Both URRBMI-insured and UEBMI-insured patients primarily obtained medications from community pharmacies (15.8% and 72.1%), followed by tertiary hospitals (2.6% and 9.5% ), with almost no utilization of secondary hospitals.

In terms of claims years, the frequency of medical insurance claims for patients increased gradually from 2018 to 2021 (0.7%, 6.5%, 26.9%, and 65.9%). The majority of claims for patients during this period occurred at community pharmacies (0.4%, 3.7%, 22.1%, and 61.7%), with a growing trend each year. Furthermore, the frequency of medical insurance claims for each year increased nearly ninefold, fourfold, and twofold compared to the previous year.

Regarding dosage forms, the frequency of medical insurance claims for oral agents (55.8%) was slightly higher than that for injection agents (44.2%). Regardless of oral or injection agents, the frequency of medical insurance claims at secondary hospitals was almost non-existent. The majority of claims for oral agents occurred at community pharmacies (55.6%), while injection agents were primarily settled at community pharmacies (32.2%) and secondary hospitals (11.9%).

### Analysis of medication costs for different patient types

From Table 4, it can be observed that according to the results of the “K-W tests”, factors such as age (*P* < 0.05), medical treatment category (*P* < 0.001), health insurance schemes (*P* < 0.001), designated type (*P* < 0.001), claims year (*P* < 0.001), and dosage form (*P* < 0.05) significantly influenced the medication costs of rare disease patients.

**Table 4:** Medication costs (in ten thousand dollars) for patients’ different demographic characteristics

Group	Designated type		Medication costs (%)	*P*
	Tertiary hospital	Community pharmacy		
**Total medication costs**	41.0(13.3%)	266.9(86.7%)	307.9(100%)	
**Age**				*P* < 0.05
1–30	3.1(1.0%)	41.9 (13.6%)	45(14.6%)	
31–40	6.9(2.2%)	41.6(13.5%)	48.5(15.7%)	
41–50	4.6(1.5%)	33.7(10.9%)	38.3(12.4%)	
51–60	7.8(2.5%)	43.6(14.2%)	51.4(16.7%)	
61–65	9.4(3.1%)	31.1(10.1%)	40.5(13.2%)	
66–70	4.2(1.4%)	38.5(12.5%)	42.7(13.9%)	
>71	5.1(1.7%)	36.6(11.9%)	41.7(13.6%)	
**Medical treatment category**				*P* < 0.001
Outpatient	0.1(0%)	1.0(0.3%)	1.1(0.3%)	
Outpatient for major diseases and outpatient for chronic diseases	3.0(1.0%)	0.1(0%)	3.1(1.0%)	
Outpatient special drugs	23.5(7.6%)	265.8(86.3%)	289.3(93.9%)	
Inpatient	14.4(4.7%)	0(0%)	14.4(4.7%)	
**Health insurance schemes**				*P* < 0.001
URRBMI	6.3(2.0%)	42.1(13.7%)	48.4(15.7%)	
UEBMI	34.7(11.3%)	224.8(73.0%)	259.5(84.3%)	
**Claims year**				*P* < 0.001
2018	1.9(0.6%)	1.4(0.5%)	3.3(1.1%)	
2019	11.5(3.7%)	12.1(3.9%)	23.6(7.6%)	
2020	17.5(5.7%)	70.8(23.0%)	88.3(28.7%)	
2021	10.1(3.3%)	182.6(59.3%)	192.7(62.6%)	
**Dosage form**				*P* < 0.05
Oral agents	0(0%)	178.4(57.9%)	178.4(57.9%)	
Injection agents	41.0(13.3%)	88.5(28.7%)	129.5(42.0%)	

Note: Since the medication expenses incurred at secondary hospitals are minimal and account for 0% of the total, they are not listed in the table

Regarding designated type, medication costs were primarily generated at community pharmacies (86.7%) and tertiary hospitals (13.3%).

In terms of age, the “51–60 years” group had the highest proportion of medication costs (16.7%), followed by the “31–40 years” group (15.7%), while the differences in medication costs among other age groups were not significant. The proportion of medication costs for patients in each age group is highest at community pharmacies, followed by tertiary hospitals, and there is no proportion at secondary hospitals.

Regarding medical treatment category, the majority of medication costs were incurred through the “outpatient specialty pharmacy” category (93.9%), followed by “inpatient” (4.7%), with a combined proportion of less than 2% for general outpatient clinics, outpatient for major diseases and outpatient for chronic diseases. This proportion aligns with the frequency of medical insurance claims proportion.

In terms of health insurance schemes, medication costs accounted for 84.3% for patients covered by URRBMI, while patients covered by UEBMI accounted for only 15.7% of the medication costs.

In terms of dosage form, medication costs for oral agents (57.9%) were higher than for injection agents (42.0%).

Looking at claims years, medication costs showed an overall increasing trend from 2018 to 2021. During the 2018–2019 period, there was a significant increase in medication costs.

### Trends in DDDs, annual drug cost, and DDDc for orphan drugs from 2018 to 2021 (average)

In this study, we first calculated the DDDs, annual drug cost, and DDDc for each drug per year. Since the number of drugs included each year varies, the average values of DDDs, annual drug cost, and DDDc for the drugs involved in each year were taken to more accurately measure the overall drug utilization each year and eliminate the influence of quantity differences on the results.

**Fig. 1:**
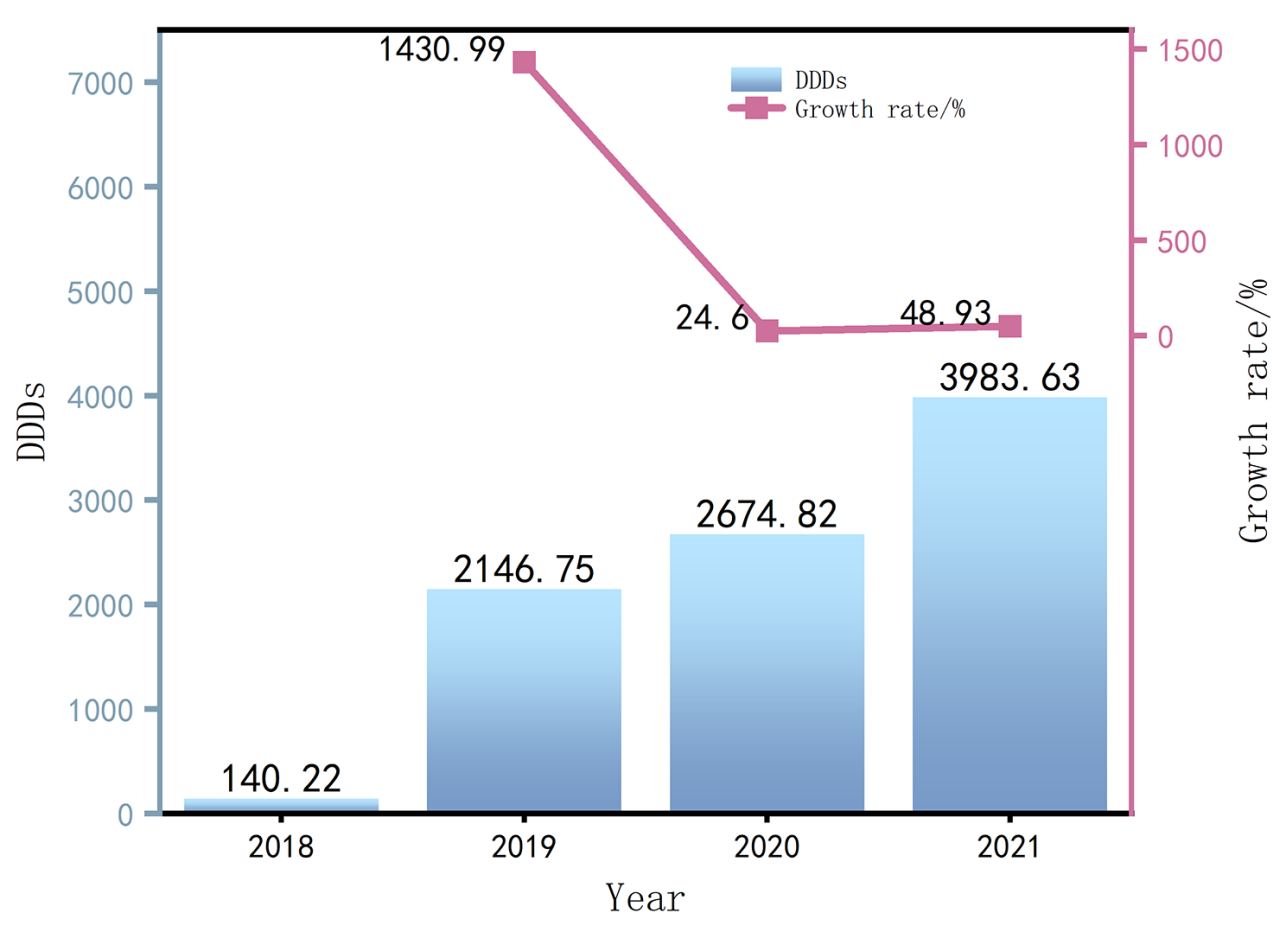
DDDs and magnitude changes of orphan drugs from 2018 to 2021 (Average)

**Fig. 2:**
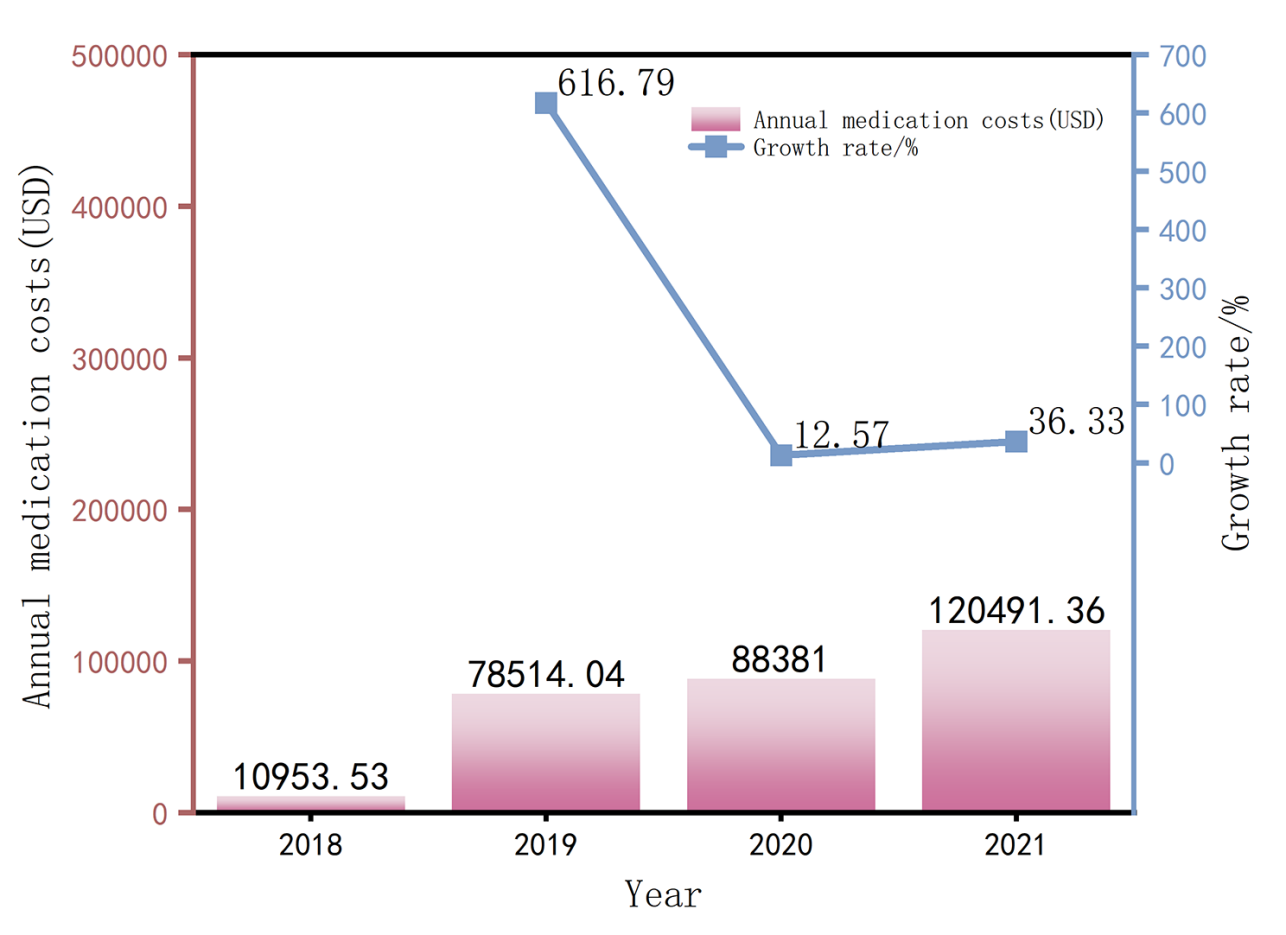
Annual medication costs (USD) and magnitude changes of orphan drugs from 2018 to 2021 (Average)

**Fig. 3:**
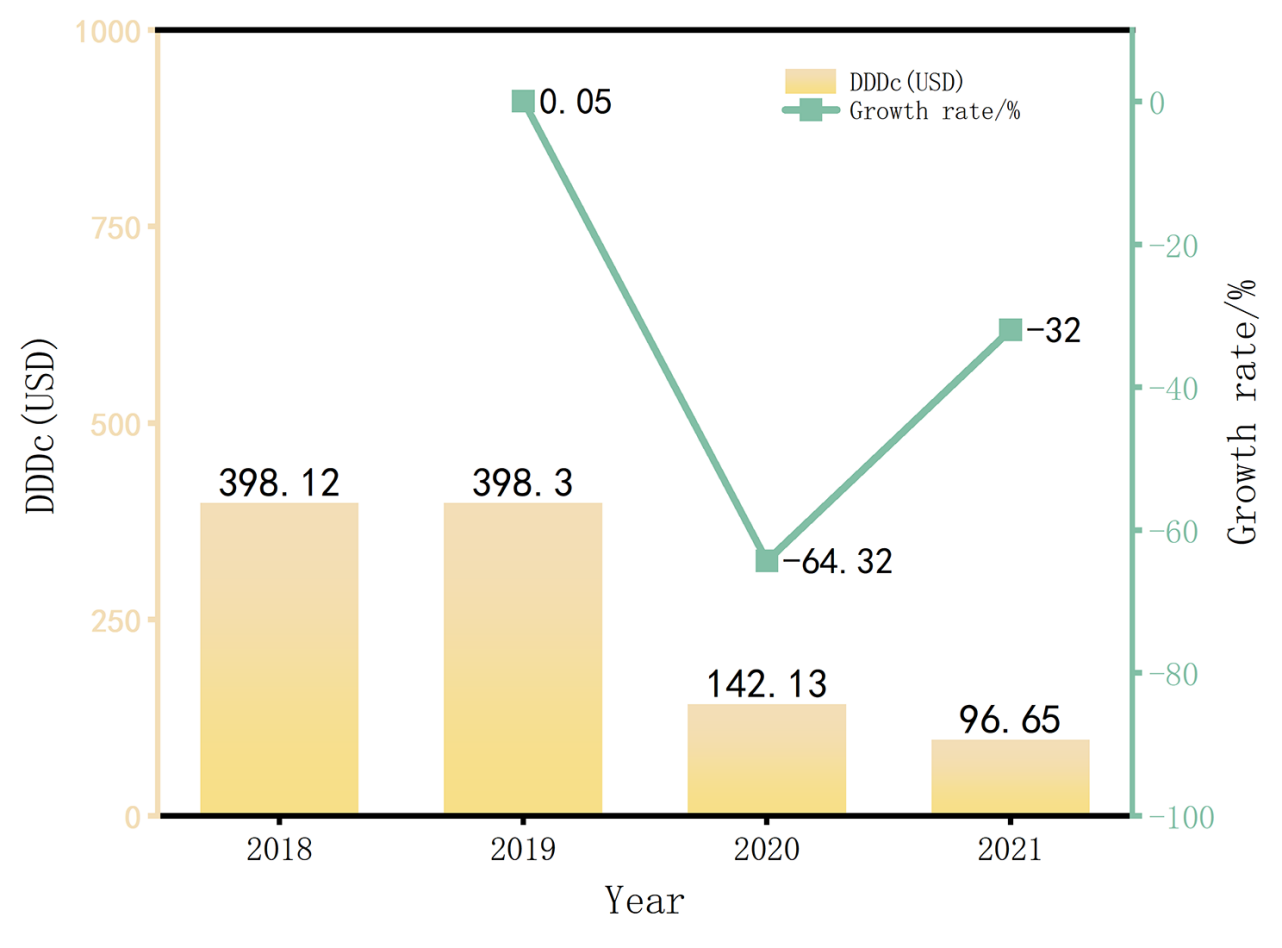
DDDc and magnitude changes of orphan drugs from 2018 to 2021 (Average)

It can be seen from Figs. 1, 2 and 3 that the average DDDs of 16 orphan drugs in a certain city from 2018 to 2021 show a year-on-year increasing trend. This indicates that the frequency of orphan drug usage has rapidly increased in the past few years, and the accessibility of orphan drugs has significantly improved. Among them, the increase in DDDs in 2019 was as high as 1430.99%, which may be due to the addition of 7 orphan drugs to the NRDL in 2019, as there were fewer rare diseases included before that. The average annual drug cost for the 16 orphan drugs also showed an increasing trend from 2018 to 2021, indicating that the growth in usage has driven the increase in drug costs after their inclusion in the NRDL. The average DDDc for the 16 orphan drugs from 2018 to 2021 showed an overall declining trend, indicating that the treatment cost per day for using a certain orphan drug has become lower over time, and the affordability of orphan drugs has significantly improved.

### Affordability of orphan drugs in a certain city from 2018 to 2021

From Table 5, it can be seen that before medical insurance coverage, the annual treatment costs of 9 out of 16 orphan drugs exceeded the CHE threshold for urban residents, and the treatment costs of 15 drugs exceeded the CHE threshold for rural residents. However, after medical insurance coverage, none of the drugs required out-of-pocket expenses that exceeded the CHE threshold for urban residents. For patients in rural areas, after medical insurance coverage, only half of the drugs no longer constituted CHE compared to before reimbursement. Regardless of whether it was before or after medical insurance coverage, the orphan drug with the best affordability was Edaravone, as its multiple of CHE was significantly lower than the other 15 drugs. For urban residents, the least affordable drugs were Recombinant Human Interferon Beta-1b and Fingolimod, with a multiple of CHE of 2.51 before medical insurance coverage. For rural residents, the least affordable drug was Recombinant Human Interferon Beta-1b, with a multiple of CHE reaching 6.75 before medical insurance coverage and 2.03 after medical insurance coverage.

Among the four medications used to treat idiopathic pulmonary arterial hypertension (IPAH) - Bosentan, Riociguat, Macitentan, and Selexipag - Bosentan has the highest affordability, while Selexipag has the lowest affordability. In the case of multiple sclerosis (MS), among the four medications - Siponimod, Teriflunomide, Fingolimod, and Recombinant Human Interferon Beta-1b - Siponimod has the highest affordability, while Recombinant Human Interferon Beta-1b has the lowest affordability. By comparing these eight medications, we can conclude that the cost-effectiveness ratio (CHE) for the four IPAH medications is lower than that of the four MS medications. This indicates that the medications for IPAH have better affordability compared to those for MS.

**Table 5:** Affordability of orphan drugs in a certain city before and after medical insurance coverage

Serial number	Generic name of drugs	Catastrophic health expenditure multiple before medical insurance coverage		Catastrophic health expenditure multiple after medical insurance coverage	
		Urban	Rural	Urban	Rural
1	Edaravone	0.03	0.08	0.00	0.01
2	Deferasirox	0.60	1.58	0.17	0.43
3	Bosentan	0.64	1.68	0.19	0.50
4	Recombinant human coagulation factor VIIa	0.75	2.00	0.07	0.20
5	Vedolizumab	0.79	2.06	0.24	0.62
6	Infliximab	0.83	2.18	0.18	0.46
7	Riociguat	0.89	2.35	0.27	0.70
8	Macitentan	1.22	3.21	0.37	0.96
9	Nintedanib	1.32	3.46	0.40	1.04
10	Ruxolitinib	1.85	4.87	0.56	1.46
11	Selexipag	1.85	4.88	0.56	1.31
12	Siponimod	2.02	5.29	0.61	1.59
13	Teriflunomide	2.40	6.32	0.72	1.90
14	Octreotide acetate microspheres	2.43	6.49	0.73	1.95
15	Fingolimod	2.51	6.58	0.75	1.97
16	Recombinant human interferon beta-1b	2.51	6.75	0.75	2.03

## Discussion

According to the analysis of DDDs, annual drug costs, and DDDc data for orphan drugs in this city from January 2018 to December 2021, the following conclusions can be drawn: Firstly, the average DDDs of orphan drugs show a year-on-year increasing trend, indicating an improvement in the accessibility of orphan drugs over time. The significant growth rate also suggests that prior to being covered, the medication needs of most patients were difficult to meet. Secondly, the annual drug costs for individual medications also showed a year-on-year increasing trend. This indicates that although pharmaceutical companies significantly reduced prices after the drugs were included in the NRDL, the expansion of the drug market and the increasing number of users have led to a “win-win” situation for both patients and pharmaceutical companies [21]. Lastly, the average DDDc of orphan drugs shows an overall decreasing trend, indicating a gradual improvement in the affordability of orphan drugs. However, the DDDc still remains at a relatively high level, indicating that even though the prices of nationally negotiated drugs have significantly decreased, they are still relatively expensive, and patients continue to face a heavy medication burden and economic pressure. Therefore, it is recommended to improve the comprehensive assistance system for patients with rare diseases and their families, clarify the roles and functions of charitable organizations and commercial insurance, and establish a rare disease high-value drug protection system led by the government with multiple stakeholders sharing the costs and risks. This will further enhance the level of medical security for rare disease patients [22].

From the perspective of medication costs, they are mainly incurred in community pharmacies and tertiary hospitals, while secondary hospitals have almost none. These findings reflect that the accessibility of orphan drugs in tertiary hospitals is significantly higher than that in primary and secondary hospitals. This may be because the diagnosis and treatment of rare diseases are difficult, and primary healthcare institutions are almost unable to provide diagnosis and treatment for patients. Moreover, under the current structure of tertiary hospitals, primary healthcare institutions have not yet played the optimal role in gatekeeping and referral [21]. Therefore, it is recommended to improve the clinical screening and diagnosis and treatment capabilities of medical staff in primary healthcare institutions for rare diseases, make reasonable use of the referral function of medical alliances, and avoid delays in patient conditions due to insufficient diagnostic and treatment capabilities [23].

In a certain city, there has been a significant increase in both the number of patients and the amount spent on purchasing orphan drugs from community pharmacies. Additionally, the settlement of orphan drug payments in this city is primarily associated with the “outpatient special drugs” healthcare delivery channel, while the majority of drug expenses are concentrated in community pharmacies. Considering this, we verified that during the data analysis period, apart from the implementation of the “dual-channel” policy, there were no other policy changes that could explain the rise in the number of patients purchasing orphan drugs. Moreover, rare diseases have a low incidence rate and are unlikely to undergo significant changes in the short term. The high financial burden imposed by expensive orphan drugs is greatly influenced by the coverage policies. Therefore, we believe that the implementation of the “dual-channel” policy can reasonably be considered as a significant factor contributing to the increase in the number of patients. These two phenomena collectively indicate that the “dual-channel” policy in this city is functioning effectively, with a well-established drug supply management mechanism, and has further improved the accessibility of medication for insured patients. The implementation of the “dual-channel” policy also means increased difficulty in the supervision of medical insurance funds, so it is necessary to strengthen the supervision of medical insurance funds. Furthermore, it is recommended to improve the supply mechanism and process of drugs in retail pharmacies, strictly monitor the storage conditions of drugs in community pharmacies and the delivery capabilities of pharmacies, achieve traceable monitoring of drug circulation and connection links, and ensure the safe use of drugs [24].

The frequency of medical insurance claims and medication costs of UEBMI-insured urban employees in a certain city are approximately five times higher than those of URRBMI-insured rural residents, indicating a significant disparity. This suggests that there are huge differences in the reimbursement benefits of different insured patients. Due to the high prices of orphan drugs and the heavy burden of treatment costs on patients, some patients may still give up treatment even with medical insurance reimbursement due to the difficulty of bearing high treatment expenses. It is recommended that the healthcare insurance department dynamically adjust the reimbursement ratio based on the actual situation of patients to effectively alleviate the burden of medication costs.

From the perspective of the annual treatment cost being a multiple of “CHE” for residents, the probability of rural residents incurring CHE due to rare diseases is greater than that of urban residents, showing significant urban-rural disparities, which is consistent with other research findings [25, 26]. Therefore, more attention should be paid to the disease burden of rural residents. Although the implementation of the NDPN policy has to some extent alleviated the burden of medication expenses for patients, the level of protection is still limited, as similar trends have been observed in previous research findings [27, 28]. The rare disease protection system should classify and manage the extremely vulnerable population, set different deductibles based on household income, and establish equal treatment based on disease or treatment needs [21].

This study has several limitations. Due to the limited number and variety of drugs in this database and the relatively short duration of drug implementation, there may be errors in the research results. Additionally, data collection was restricted to a single city, which limits the generalizability of the findings. Furthermore, the data for this study were obtained from the health insurance claims data and did not include information on drug purchases made by individuals who were not reimbursed or who paid for the drugs entirely out of pocket. Finally, due to the lack of data from the pre-implementation period, this study was unable to assess the impacts of the negotiation policy on the utilization and affordability of the rare disease drugs.

## Conclusion

The health insurance reimbursement policy in a tier-one city has been operating well, and the utilization frequency of orphan drugs has gradually increased. However, the burden of disease still remains significant. Improving the affordability of orphan drugs is a complex and long-term process that requires concerted efforts from the government, healthcare institutions, and various sectors of society. It necessitates the establishment of comprehensive policy measures to provide better support and protection to patients, aiming to achieve sustainable development in the field of rare diseases.

## Data Availability

The data that support the findings of this study are available from the Healthcare Security Administration. However, restrictions apply to the availability of these data, which we used under licence for the current study; thus, they are not publicly available. However, the data are available from the authors upon reasonable request and with the permission of the Healthcare Security Administration.

## Abbreviations

DDDs: Defined daily doses
DDDc: Defined daily drug cost
CHE: Catastrophic health expenditure
NRDL: National reimbursement drug list
NDPN: National drug price negotiation
NHSA: National Healthcare Security Administration
URRBMI: Urban-rural residents’basic medical insurance
UEBMI: Urban employees’basic medical insurance
K-W: Kruskal-Wallis
IPAH: Idiopathic pulmonary arterial hypertension
MS: Multiple sclerosis
